# Multimodal MRI Assessment of neuroprotective effects of Ofatumumab on brain structure, function, and clinical correlates in Relapsing Multiple Sclerosis

**DOI:** 10.3389/fneur.2026.1738469

**Published:** 2026-02-20

**Authors:** Jing An, Xiaoshuang Wang, Zhaoshi Zheng, Yingyu Zhang, Di Wang, Yaxin Qu, Qiurong Yang, Shuai Wang, Xuemei Han

**Affiliations:** 1 Department of Neurology, China-Japan Union Hospital of Jilin University, Changchun, China; 2 Medical Department, The Sixth Hospital of Changchun, Changchun, China

**Keywords:** disease modifying therapy, functional connectivity, Multiple Sclerosis, neuroimaging, Ofatumumab

## Abstract

Relapsing Multiple Sclerosis (RMS) is characterized by neuroinflammation and neurodegeneration, leading to disability. Ofatumumab (OFA), an anti-CD20 monoclonal antibody, has shown promise as a disease-modifying therapy. This study aimed to assess the neuroprotective effects of OFA in RMS using multimodal magnetic resonance imaging (MRI). We conducted a retrospective cohort study comparing 16 RMS patients receiving OFA for 1 year (Treatment Group, TG) with 8 treatment-naïve patients (No-Treatment Group, NTG). Participants underwent 3T MRI scans, including 3D T1-weighted, diffusion tensor imaging (DTI), and resting-state functional MRI (rs-fMRI). Clinical outcomes were measured using the Expanded Disability Status Scale (EDSS), timed 25-Foot Walk (T25FW), and cognitive assessments. Results showed significant improvements in motor function and anxiety in the TG, alongside increased white matter integrity and stable cognition. Notably, TG patients exhibited enhanced functional connectivity between the thalamus and cortex, as well as increased fractional anisotropy (FA) in key white matter tracts. In contrast, the NTG displayed gray matter atrophy. These preliminary findings suggest that OFA treatment may preserve brain structure and function in RMS, with potential neuroprotective effects mediated, through thalamocortical network modulation and white matter restoration.

## Introduction

Multiple Sclerosis (MS) is a chronic autoimmune disorder of the central nervous system characterized by inflammation, demyelination, reactive astrogliosis, and neuronal loss (1, 2). It predominantly occurs in individuals aged 29–39 years, with a higher prevalence in women (3). The pathogenesis of MS involves complex interactions between genetic susceptibility and environmental factors, such as Epstein-Barr virus exposure, vitamin D deficiency (4). Autoreactive T cells breach the blood-brain barrier, target myelin and oligodendrocytes, and drive neuro inflammation and neuro degeneration (5).

Magnetic Resonance Imaging (MRI) plays a central role in the diagnosis and monitoring of MS. Conventional sequences, includingT2-weighted, gadolinium-enhanced T1-weighted, and fluid-attenuated inversion recovery (FLAIR) imaging, are used to detect lesions and atrophy. Advanced techniques such as diffusion tensor imaging (DTI) and resting-state functional MRI (rs-fMRI) provide quantitative measures of microstructural integrity and functional connectivity (FC) (6, 7). The thalamus, a key deep gray matter (GM) nucleus, frequently exhibits atrophy that correlates with disability progression (6), while aberrant thalamocortical FC is implicated in cognitive and motor dysfunction (8).

Disease-modifying therapies (DMTs) are essential for reducing annualized relapse rates and slowing disability accumulation in MS (9). Ofatumumab (OFA), a fully human anti-CD20 monoclonal antibody, depletes B cells that contribute to MS immunopathology (10) and has demonstrated favorable efficacy and safety profiles compared to several existing treatments (11). Modern MS management strives to achieve the “No Evidence of Disease Activity” standard, which integrates clinical outcomes (relapses, disability progression), MRI metrics (new lesions, brain atrophy), and molecular biomarkers (12–14). Multimodal MRI, particularly rs-fMRI-derived metrics [e.g., Regional Homogeneity (ReHo), FC] and DTI parameters [e.g., Fractional Anisotropy (FA), Mean Diffusivity (MD)], provides sensitive *in vivo* markers for monitoring axonal integrity and adaptive functional reorganization (15–17). Nevertheless, evidence regarding OFA's neuroprotective effects, particularly its influence on thalamic structure-function relationships and white matter integrity as assessed through integrated multimodal MRI, remains limited. This gap hinders personalized therapeutic optimization.

Given the pivotal role of thalamic structure and function in MS therapy and the neuroprotective potential of OFA, this study employs multimodal MRI—including resting-state FC, ReHo, and DTI —to longitudinally evaluate OFA's effect on thalamocortical network reorganization, white matter microstructural restoration, and gray matter volume preservation in relapsing MS (RMS) patients. Furthermore, we explore correlations between imaging-derived metrics and improvements in motor and cognitive function, aiming to provide neuroimaging evidence that elucidates OFA's disease-modifying mechanisms and supports advances in personalized clinical management.

## Methods

### Study participants

This retrospective cohort study enrolled RMS patients from the China-Japan Union Hospital of Jilin University between August 2019 and January 2025. All patients were diagnosed according to the 2017 revised McDonald criteria. Inclusion criteria: (1) absence of other psychiatric or neurological disorders; (2) right-handedness; (3) ability to cooperate with MRI scanning and clinical assessments; (4) age < 60 years (to minimize confounding effects from age-related atrophy); (5) matching of age, sex, and education level between the two groups; (6) no prior exposure to any DMTs; (7) documented clinical relapse within 6 months prior to enrollment. (8) All patients in the TG initiated Ofatumumab treatment within 1 month prior to baseline MRI scanning. Exclusion criteria: (1) history of comorbid neurological or neuropsychiatric disorders; (2) severe visual or hearing impairment that could affect neuropsychological assessment; (3) contraindications to MRI; (4) history of substance abuse or alcohol dependence; (5) severe cardiopulmonary disease or significant functional impairment/poor general health; (6) excessive head motion artifacts affecting MRI data quality; (7) incomplete clinical data or missing key information. A total of 32 RMS patients were initially screened. Among them, eight patients were excluded: three due to MRI contraindications, two due to excessive head motion (≥1.5 mm translation or ≥1.5° rotation), two due to comorbid neurological disorders, and one due to incomplete clinical data. The remaining 24 patients were assigned to the Treatment Group (TG, *n* = 16) or No-Treatment Group (NTG, *n* = 8) based on treatment status. All participants were followed for 1 year.

### Clinical data and scale assessments

Demographic information including age, sex, education years, and disease duration was collected. In the TG, clinical assessments were conducted within 24 h prior to MRI scanning, including the Montreal Cognitive Assessment (MoCA) (18), Hamilton Anxiety Rating Scale (HAMA) (19), Hamilton Depression Rating Scale (HAMD) (20), Modified Fatigue Impact Scale (MFIS), Timed 25-Foot Walk (T25FW), 12-item Multiple Sclerosis Walking Scale (MSWS-12), and Expanded Disability Status Scale (EDSS) (21).

### MRI acquisition

All MRI data were acquired using a Siemens 3.0T Trio Tim scanner (Erlangen, Germany). The imaging protocol included: (1) rs-fMRI: TR = 2,000 ms, TE = 30 ms, FA = 90°, slices = 36, slice thickness (ST) = 4.0 mm, gap = 0.0 mm, voxel size = 3 × 3 × 3 mm^3^, matrix = 64 × 64, FOV = 210 × 210 mm^2^; (2) 3D-T1WI: TR = 2,300 ms, TE = 2.32 ms, ST = 1.0 mm, gap = 0 mm, FOV = 240 × 240 mm^2^; (3) DTI: TR = 3,900 ms, TE = 114 ms, slices = 25, ST = 4.0 mm, gap = 1.2 mm, FOV = 220 × 220 mm^2^ (Diffusion gradients: 64 directions, *b* = 1,000 s/mm^2^; 1 *b* = 0 image). Total scan time ranged from 40 to 60 min. Participants with head motion >1.5 mm or rotation >1.5° were excluded.

### MRI data preprocessing

#### rs-fMRI preprocessing

Preprocessing was performed using the REST toolkit (http://www.restfmri.net) based on MATLAB R2016b. Steps included slice timing correction, realignment, spatial normalization to MNI space, spatial smoothing (FWHM = 6 mm), and nuisance regression (Friston 24-parameter motion, white matter, CSF signals). Bandpass filtering (0.01–0.08 Hz) was applied.

#### FC analysis

(1) ROI-based FC (Thalamus): Thirty-one thalamic subregions defined by the AAL3 atlas were used as regions of interest (ROIs). The mean BOLD time series from each ROI were correlated with every other brain voxel using Pearson correlation to generate thalamocortical FC maps, which were then converted to *z*-values using Fisher's *z*-transformation. (2) Voxel-wise FC (VWFC): Whole-brain FC maps were constructed by correlating the mean global signal time series with the time series of each individual voxel. Resulting correlation maps were also Fisher *z*-transformed.

#### DTI preprocessing

DTI data were preprocessed using PANDA toolbox (http://www.nitrc.org/projects/panda/). Steps included Voxel-wise group analysis of FA data was performed using Tract-Based Spatial Statistics (TBSS) implemented in FSL.

#### Voxel-based morphometry (VBM)

Structural T1-weighted images were processed using the CAT12 toolbox (http://www.neuro.uni-jena.de/cat/) within SPM12 (https://www.fil.ion.ucl.ac.uk/spm/). Steps included tissue segmentation, spatial normalization, modulation (to preserve volume), and smoothing (Gaussian kernel with FWHM = 8 mm) to produce gray matter volume (GMV) maps.

### Statistical analysis

#### Demographics and clinical scales

Analyses were conducted using SPSS 25.0. Group differences in demographics were assessed using Fisher's exact test (categorical variables, e.g., sex) and independent samples *t*-tests (continuous variables, e.g., age, education, disease duration). Within-group changes in clinical scales were evaluated using paired *t*-tests for normally distributed data and Wilcoxon signed-rank tests for *P* < 0.05 was non-normally distributed data; the latter are presented as median [interquartile range].

#### Imaging metrics (ReHo, fALFF, VWFC, FC, GMV)

Longitudinal within-group analyses (paired *t*-tests) and cross-sectional between-group comparisons (two-sample *t*-tests) were performed in SPM12 (MATLAB R2016a), with appropriate covariates included. Statistical maps were thresholded at voxel-level *P* < 0.001 (uncorrected) and cluster-level *P* < 0.05 (FDR-corrected).

#### DTI/TBSS

Within-group longitudinal changes in DTI metrics were analyzed using paired *t*-tests in SPSS. Between-group differences in FA at follow-up were assessed using TBSS in FSL with two-sample *t*-tests, employing threshold-free cluster enhancement (TFCE) correction with *P* < 0.05.

#### Imaging-clinical correlations

In the TG, we performed exploratory correlations between follow-up imaging metrics (FA in significant tracts, FC strength of significant connections) and changes in clinical scores (e.g., EDSS, MoCA). To control for the increased risk of false positives due to multiple comparisons, we have applied False Discovery Rate (FDR) correction to these specific correlation analyses. The results reported in the manuscript are those that survived the FDR correction (*P*_FDR < 0.05).

## Results

### Demographics and clinical scores

No significant differences were observed between the TG (*n* = 16) and the NTG (*n* = 8) in terms of age, sex, education, disease duration, EDSS, T2 lesion volume, or in head motion during rs-fMRI acquisition at baseline (all *P* > 0.05; Table 1), indicating that the two groups were well-matched in both demographic characteristics and disease severity. Notably, all patients in both groups were DMT-naïve and had experienced a clinical relapse within 6 months prior to enrollment, ensuring comparable disease activity status at study entry.

**Table 1:** Baseline demographic and clinical characteristics.

**Characteristic**	**TG (*n* = 16)**	**NTG (*n* = 8)**	***P*-value**
Sex (male/female)	6/10	2/6	0.667^†^
Age (years)	42.3 ± 10.49	38.1 ± 5.67	0.218^*^
Education (years)	9.9 ± 3.24	10.9 ± 2.75	0.463^*^
Disease duration (months)	55.0 ± 62.70	48.4 ± 41.58	0.792^*^
Baseline EDSS	2.75 [1.75, 4.00]	2.00 [1.50, 2.50]	0.089^‡^
Mean framewise displacement (FD, mm)	0.07 ± 0.02	0.08 ± 0.04	0.17^*^
T2 lesion volume (cm^3^)	8.08 [4.54, 11.44]	5.61 [3.76, 8.25]	0.214^‡^
Time on OFA prior to study (months)	0.6 ± 0.24	-	-

Data are presented as Mean ± standard deviation or median [interquartile range].^*^Independent samples *t*-test.

^†^Fisher's Exact Test.

^‡^Mann-Whitney *U*–test.

Clinical scores at baseline and 1-year follow-up for both groups are summarized in Table 2. In the TG, significant improvements were observed after 1 year of OFA treatment, including reductions in EDSS (*P* = 0.029), T25FW (*P* = 0.048), MSWS-12 (*P* = 0.023), and HAMA (*P* = 0.022) scores. Scores on the MoCA, HAMD, and MFIS remained stable, with no significant changes from baseline (all *P* > 0.05). In contrast, patients in the NTG exhibited significant worsening in EDSS (*P* = 0.019) and HAMA (*P* = 0.039) scores at follow-up. No significant changes were observed in MoCA, HAMD, MFIS, or other assessed measures (all *P* > 0.05) (Table 2).

**Table 2:** Clinical scores at baseline and one-year follow-up.

**Scale**	**Treatment group (TG**, ***n*** = **16)**				**No-treatment group (NTG**, ***n*** = **8)**			
	**Baseline**	**Follow-up**	***P*** **-value**	**Cohen's** ***d*****/*****r***	**Baseline**	**Follow-up**	***P*** **-value**	**Cohen's** ***d*****/*****r***
MoCA	25.53 ± 3.56	24.93 ± 3.67	0.108	−0.17	26.00 ± 2.51	25.88 ± 2.30	0.885	−0.08
HAMA	9.38 ± 8.03	7.50 ± 7.31	0.022	−0.64	6.50 ± 4.54	9.62 ± 5.50	0.039	1.09
HAMD	8.00 [4.25, 18.00]	6.50 [3.25, 18.50]	0.364	0.24	6.38 ± 4.90	7.38 ± 5.80	0.374	0.35
MFIS	20.00 [12.00, 36.50]	18.00 [11.00, 37.50]	0.487	0.18	15.50 ± 10.90	17.50 ± 12.74	0.081	0.58
EDSS	2.75 [1.75, 4.00]	2.50 [2.00, 3.50]	0.029	0.57	2.00 [1.50, 2.50]	2.75 [2.25, 3.00]	0.019	0.86
T25FW (s)	5.93 [4.52, 7.77]	5.20 [4.35, 7.41]	0.048	0.50	-	-	-	
MSWS-12	8.00 [0.00, 34.00]	3.50 [0.00, 30.00]	0.023	0.57	-	-	-	

Data are presented as mean ± standard deviation or median [interquartile range]. Paired *t*-test was used for normally distributed data (HAMA, MoCA); Wilcoxon signed-rank test was used for non-normally distributed data (HAMD, MFIS, EDSS, T25FW, MSWS-12).

MoCA, Montreal Cognitive Assessment; HAMA, Hamilton Anxiety Rating Scale; HAMD, Hamilton Depression Rating Scale; MFIS, Modified Fatigue Impact Scale; EDSS, Expanded Disability Status Scale; T25FW, Timed 25-Foot Walk; MSWS-12: 12-item Multiple Sclerosis Walking Scale.

Data are presented as mean ± standard deviation or median [interquartile range]. Paired *t*-test was used for normally distributed data (HAMA, MoCA); Wilcoxon signed-rank test was used for non-normally distributed data (HAMD, MFIS, EDSS, T25FW, MSWS-12). MoCA: Montreal Cognitive Assessment; HAMA: Hamilton Anxiety Rating Scale; HAMD: Hamilton Depression Rating Scale; MFIS: Modified Fatigue Impact Scale; EDSS: Expanded Disability Status Scale; T25FW: Timed 25-Foot Walk; MSWS-12: 12-item Multiple Sclerosis Walking Scale.

### MRI results

**Figure 1:**
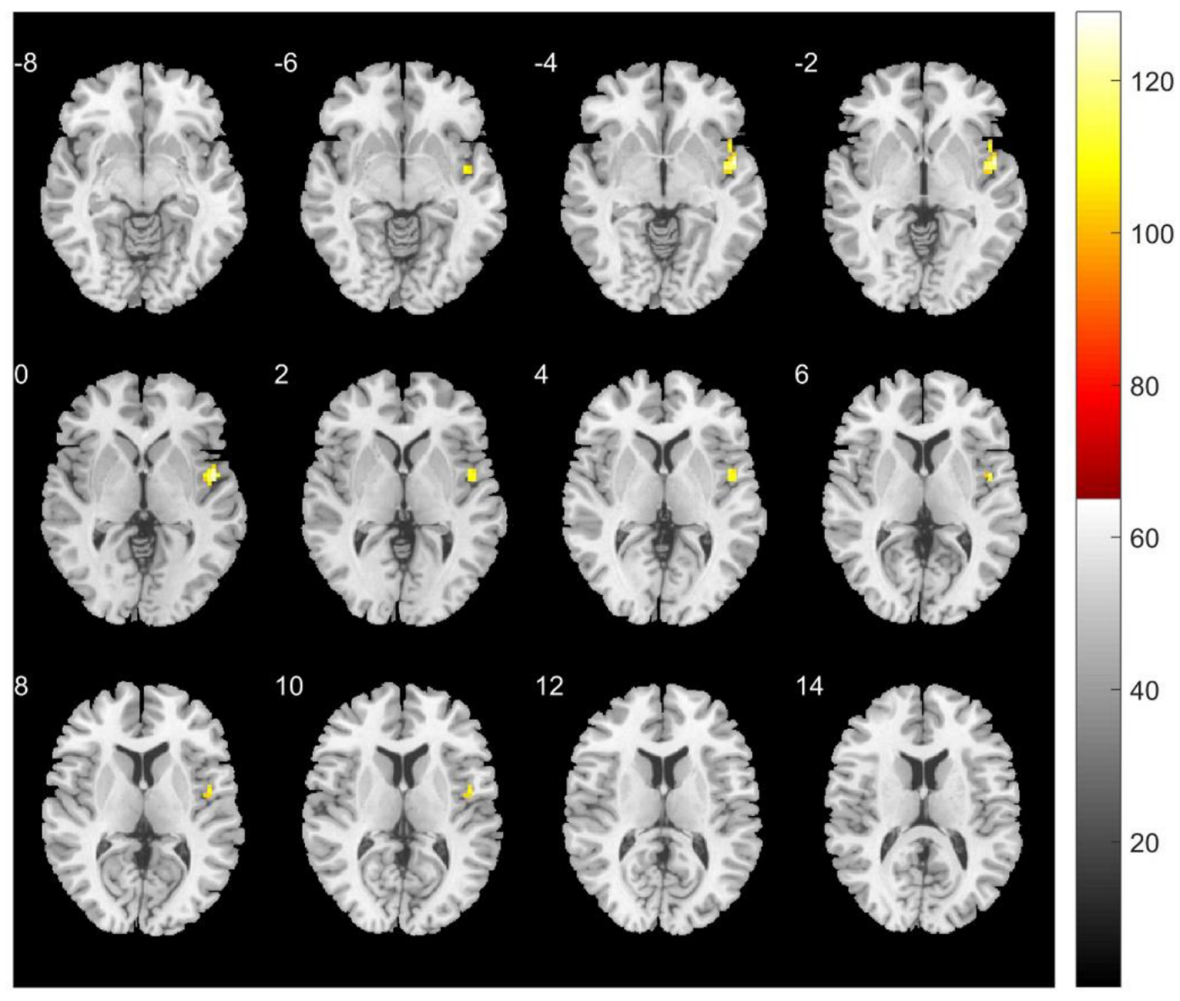
Longitudinal changes in functional connectivity between the left posterior thalamic nucleus and the whole brain in the TG. The yellow region indicates a significant increase in functional connectivity from baseline to follow-up between the left posterior lateral thalamic nucleus and the right superior temporal gyrus (*P* < 0.001, FDR corrected, voxel >42).

Significant longitudinal changes in thalamocortical FC were observed in both groups after 1 year (FDR-corrected, *P* < 0.001). In the TG, a significant increase in FC was found between the left posterior thalamic nucleus and right superior temporal gyrus (cluster size = 42 voxels; MNI peak: *x* = 48, *y* = 0, *z* = 0; *T* = 6.61) (Figure 1, Table 3). In the NTG, widespread increases in FC were detected between multiple thalamic nuclei and cortical regions, including the angular gyrus, superior parietal gyrus, precuneus, inferior frontal gyrus, medial frontal gyrus, middle temporal gyrus, and cerebellum (Table 4).

**Table 3:** Differences in functional connectivity between thalamic nuclei and other brain regions at baseline and follow-up in the TG.

**Thalamic nuclei (AAL3 template)**	**Other brain regions (AAL3 template)**	**Lump size (number of voxels)**	**MNI coordinate**			***T-*Value**
			***X***	***Y***	***Z***	
The left posterior lateral nucleus of the thalamus	Right superior temporal gyrus	42	48	0	0	6.61

MNI, Montreal Neuroscience Institute.

MNI coordinates are peak coordinates, *P* < 0.001, FDR corrected, FC values at follow-up were greater than baseline in the treatment group.

**Table 4:** Brain regions with differential functional connectivity between thalamic nuclei and other brain regions at baseline and follow-up in the NTG.

**Thalamic nuclei**	**Other brain regions**	**Number of voxels**	**MNI coordinates**			***T*-value**
			***X***	***Y***	***Z***	
Left ventral thalamic nucleus	Right angular gyrus	27	51	−60	51	7.22
Left ventral thalamic nucleus	Left superior parietal gyrus	80	−33	−72	54	9.56
Ventral nucleus of the right thalamus	Left inferior frontal gyrus	29	−12	24	45	8.67
The ventral anterior nucleus of the right thalamus	Right medial frontal gyrus	54	3	27	39	7.68
Ventrolateral nucleus of the left thalamus	The right lower lobe of the brain	43	15	−42	9	13.74
Ventrolateral nucleus of the left thalamus	Right precuneus	63	27	−72	33	11.03
Ventrolateral nucleus of the left thalamus	The right middle occipital gyrus	37	30	−60	0	10.60
Ventrolateral nucleus of the left thalamus	Left precuneus	33	−27	−69	39	8.74
Ventrolateral nucleus of the left thalamus	Left inferior frontal gyrus	52	−36	39	6	7.93
Dorsomedial nucleus of the right thalamus	The falciform gyrus of the right temporal lobe	62	33	−48	−18	14.57
Medial dorsal nucleus of the left thalamus	Right middle temporal gyrus	51	42	−75	15	11.82
Medial dorsal nucleus of the left thalamus	Left superior parietal lobe	31	−30	−66	45	11.18
Medial dorsal nucleus of the left thalamus	Right posterior cerebellum	30	0	−66	21	8.93
The left lateral geniculate body of the thalamus	Left superior frontal gyrus	39	−21	24	57	12.92
The left lateral geniculate body of the thalamus	Left superior parietal gyrus	50	−27	−81	45	10.05
The left lateral geniculate body of the thalamus	Right posterior cerebellum	79	0	−69	−27	9.22
The left lateral geniculate body of the thalamus	Right fusiform gyrus	41	27	−45	−18	9.00
The left lateral geniculate body of the thalamus	Left inferior temporal gyrus	27	−30	−48	−3	8.20

MNI, Montreal Neuroscience Institute.

MNI coordinates are peak coordinates *P* < 0.001, FDR corrected, FC values at follow-up were greater than baseline in the NTG.

In the TG, FA values were increased in the right inferior front-occipital fasciculus, uncinate fasciculus, and inferior longitudinal fasciculus (*P* < 0.05). In contrast, no significant changes in FA were detected in any white matter tracts in the NTG (Table 5).

**Table 5:** Significant changes in FA values within the TG between baseline and follow-up.

**White matter tract**	**Baseline**	**Follow-up**	***P-value***
right inferior frontal-occipital bundle	0.35 ± 0.04	0.36 ± 0.04	0.019
right inferior longitudinal fasciculus	0.36 ± 0.05	0.37 ± 0.044	0.017
right hooked fasciculus	0.33 ± 0.03	0.34 ± 0.03	0.001

FA, Fractional Anisotropy; Statistical Description.

Mean ± Standard Deviation. A *p-*value < 0.05 was considered statistically significant based on the results of the paired-sample *t*-test.

**Figure 2:**
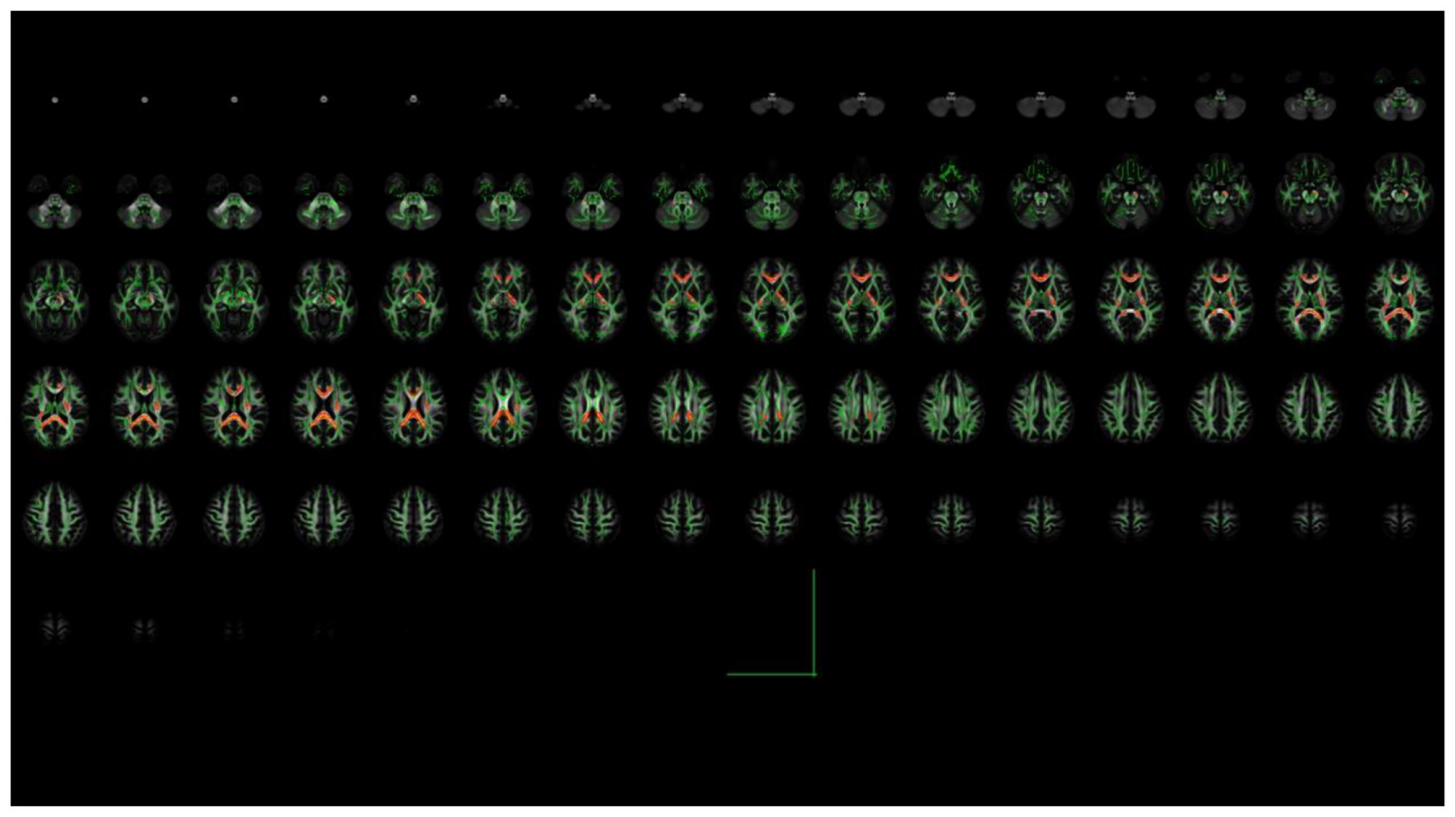
TBSS skeletal representation of FA differences between the TG and the NTG. The green background represents the mean FA skeleton. Regions highlighted in red indicate significantly higher FA values (*P* < 0.05, corrected) in the TG compared to the NTG, encompassing the body and splenium of the corpus callosum, bilateral posterior corona radiata (including optic radiations).

TBSS analysis revealed significantly higher FA values in the TG compared to the NTG at follow-up in several white matter structures (*P* < 0.05, corrected), including the corpus callosum and bilateral Radiating corona (including the optic radiation) (Table 6 and Figure 2).

**Table 6:** White matter regions with significantly higher FA values in the TG compared to the NTG at follow-up (TBSS Analysis).

**White matter fiber**	**Lump size (number of voxels)**	**MNI coordinate**			***P-*value**
		**X**	**Y**	**Z**	
corpus callosum	1538	−15	−46	18	0.015
Right posterior radiating corona	111	17	−40	28	0.041
Left posterior radiating corona including optic radiation)	23	−22	−52	9	0.049

Montreal Institute for Neuroscience, MNI coordinates represent the peak positions, *P* < 0.05, after multiple comparison corrections: the brain regions with higher FA values in the TG than those in the NTG: the corpus callosum, bilateral radiating corona (including optic radiation).

**Figure 3:**
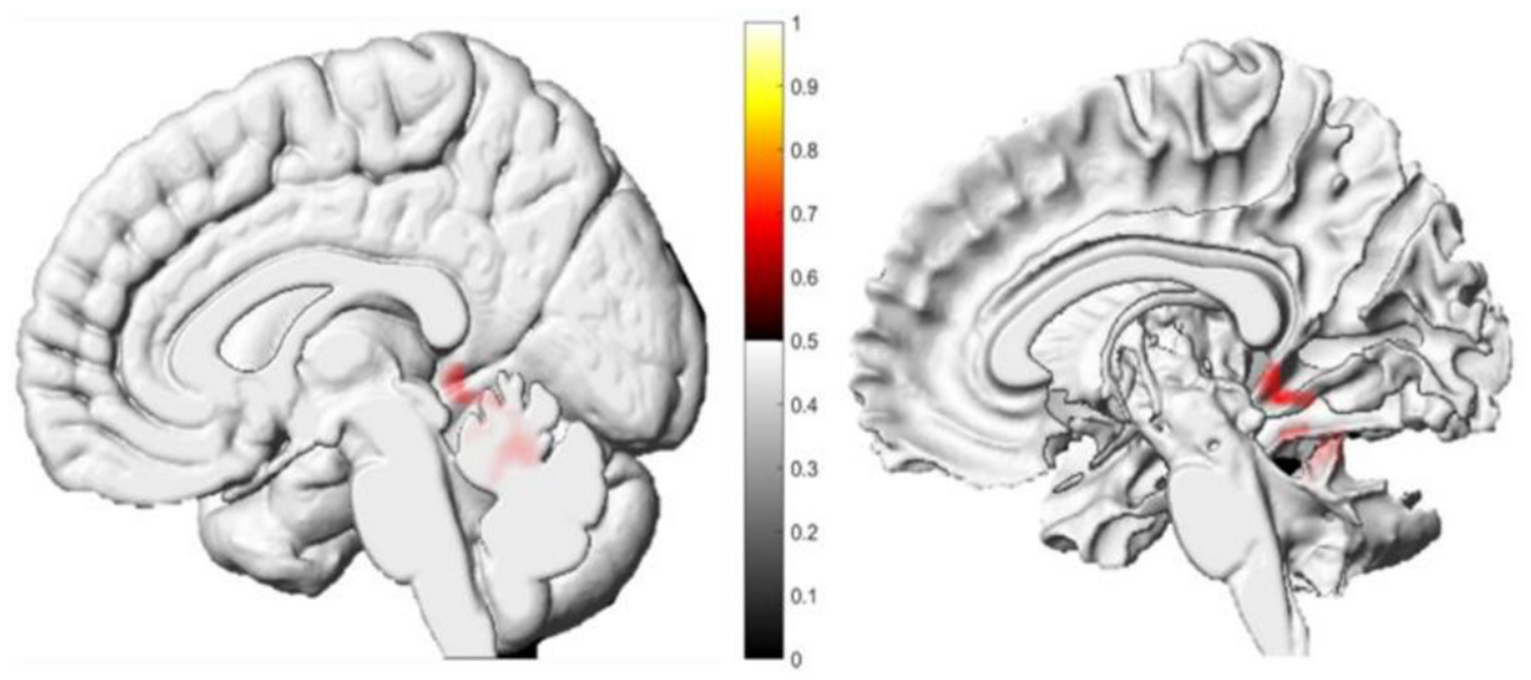
Longitudinal changes in regional gray matter volume within the NTG: baseline vs. follow-up. The red cluster indicates a significant reduction in gray matter volume at follow-up, localized to the right parahippocampal gyrus (cluster-level FDR-corrected *P* < 0.001; cluster size >329 voxels).

Voxel-based morphometry analysis revealed differential patterns of GMV change between groups. In the TG, there were no significant changes in global or regional GMV. In contrast, the NTG exhibited a significant reduction in GMV in the right parahippocampal gyrus (*P* < 0.001, FDR-corrected) (Table 7 and Figure 3).

**Table 7:** Significant reduction in gray matter volume in the NTG from baseline to follow-up.

**Brain region**	**Lump size (number of voxels)**	**MNI coordinate**			***t*-value**
		**X**	**Y**	**Z**	
right parahippocampal gyrus	329	22.5	−43.5	−6	8.7

MNI, Montreal Institute for Neuroscience; MNI coordinates represent the peak positions.

*P* < 0.001, Corrected by FDR, The gray matter volume in the NTG at follow-up was reduced compared to the baseline measurement.

### Imaging-clinical correlations

**Figure 4:**
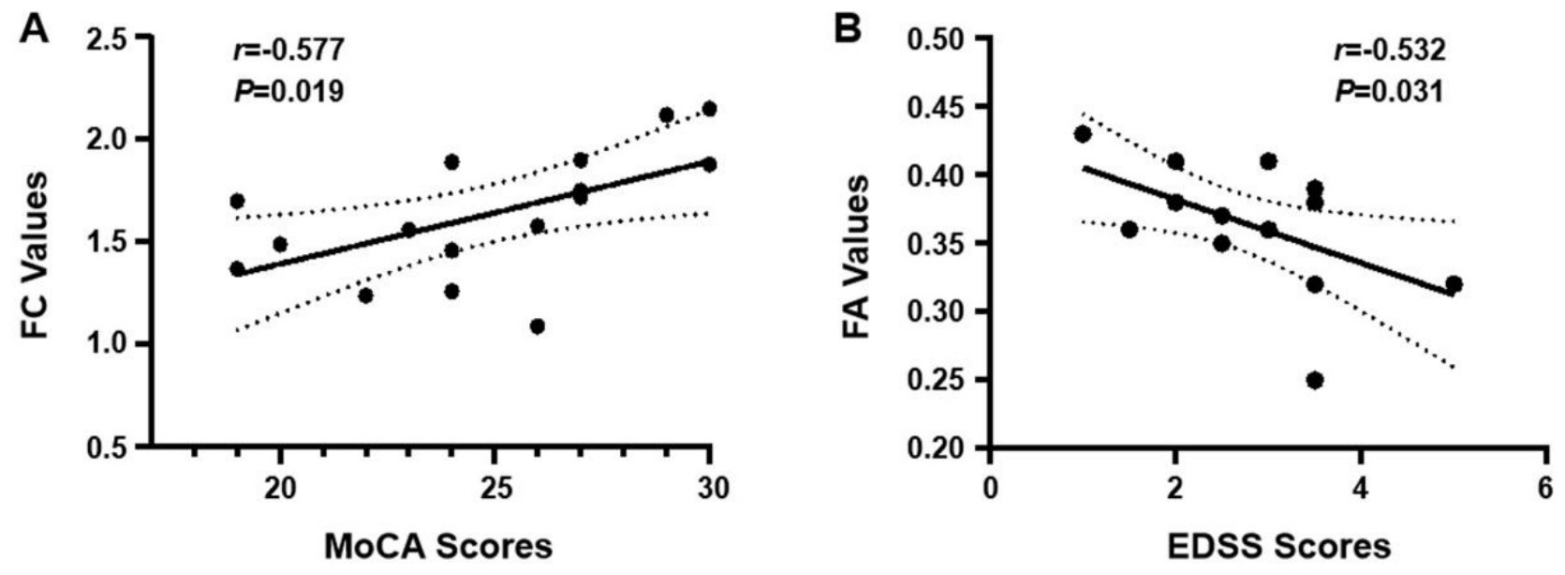
**(A)** Correlation between FC and cognitive performance in the TG. FC values between the left thalamus (posterior lateral nucleus) and the whole brain at follow-up show a significant positive correlation with MoCA scores (*r* = 0.577, *P* = 0.019). **(B)** Correlation between white matter integrity and clinical disability in the TG. FA values within the right inferior longitudinal fasciculus at follow-up are negatively correlated with EDSS scores (*r* = −0.532, *P* = 0.031).

In the TG, increased FC strength between the left thalamus and the right superior temporal gyrus at follow-up showed a significant positive correlation with MoCA scores (*r* = 0.577, *P* = 0.019, *r*^2^ = 0.33), suggesting that enhanced thalamocortical connectivity is associated with better cognitive performance (Figure 4A). In the TG, the increase in FA within the right inferior longitudinal fasciculus at follow-up was significantly negatively correlated with EDSS scores (*r* = −0.532, *P* = 0.031, *r*^2^ = 0.28), indicating that microstructural improvement in this tract is associated with reduced disability (Figure 4B).

## Discussion

This first longitudinal multimodal MRI study provides evidence supporting the neuroprotective effects of OFA on thalamocortical network reorganization, white matter microstructural restoration, and gray matter preservation in RMS. The principal findings are as follows.

### OFA modulates thalamocortical functional connectivity

Thalamic neurodegeneration is a pivotal contributor to neurological disability in MS (22, 23). Our results indicate that the NTG exhibited widespread increases in thalamocortical FC, particularly involving the angular gyrus, superior parietal lobule, and precuneus (24, 25), consistent with previously reported compensatory neural reorganization (16, 26). In contrast, the TG showed a selective enhancement of FC between the left thalamus and right superior temporal gyrus. Notably, this region-specific increase in FC was positively correlated with improved cognitive performance (MoCA score: *r* = 0.577, *P* = 0.019). These findings support the compensatory plasticity hypothesis, suggesting that OFA may help regulate pathological thalamocortical hyper connectivity (27), thereby promoting cognitive network stability. This provides novel evidence that B-cell depletion therapy may indirectly facilitate neuroadaptive processes. However, the absence of a healthy control group limits our ability to conclude whether this regulation normalizes connectivity to physiological levels; future studies with HC are needed to validate this.

### OFA facilitates white matter microstructural restoration

Analysis of DTI metrics revealed significantly increased FA (*P* < 0.05) in the right inferior fronto-occipital fasciculus (IFOF), uncinate fasciculus (UF), and inferior longitudinal fasciculus (ILF) following OFA treatment. The negative correlation between ILF FA and EDSS (*r* = –0.532, *P* = 0.031) is consistent with the established role of temporal limbic tract integrity in cognitive-motor function (21), suggesting improved axonal coherence. Furthermore, TBSS demonstrated significantly higher FA values (*P* < 0.05) in the corpus callosum, corona radiata, and optic radiation in the TG compared to the NTG at follow-up, highlighting the OFA's protective efficacy in critical WM pathways (28). Importantly, this microstructural restoration contrasts with the FA reductions observed under natalizumab therapy (20, 29), underscoring the distinct neuroprotective profile of OFA.

### OFA attenuates gray matter atrophy progression

Significant atrophy was observed in the right parahippocampal gyrus (*P* < 0.001) in the NTG, whereas the TG exhibited preservation of global GMV. Although DMTs generally demonstrate limited efficacy in reducing brain atrophy (30), our results suggest that OFA may help maintain structural integrity in limbic circuits by suppressing active neurodegeneration (31, 32). This preservation of limbic GM, together with thalamic protection, likely contributes to cognitive stability (33, 34).

We note that Ofatumumab is a fully human anti-CD20 monoclonal antibody whose primary target is CD20-expressing peripheral B lymphocytes. Administered subcutaneously, it effectively depletes peripheral B cells, thereby reducing the infiltration of pro-inflammatory B cells into the central nervous system and their antibody secretion. It also indirectly modulates the intracranial immune environment by depleting CD20? T cells, inhibiting T-cell migration, and reshaping peripheral immune homeostasis. Current evidence suggests that large-molecule antibodies such as ofatumumab mainly exert their effects through peripheral immunomodulation, with limited ability to cross the intact blood–brain barrier. Therefore, the observed improvement in thalamocortical functional connectivity is more likely attributable to the suppression of peripheral inflammation, reduction in CNS infiltration of B and T cells, and decreased release of related inflammatory factors, which collectively foster a microenvironment conducive to neuroplastic recovery, rather than direct drug entry into brain tissue.

## Limitations and future perspectives

Several limitations of this study should be acknowledged. First, the small sample size (TG: *n* = 16; NTG: *n* = 8) is a major limitation that may compromise statistical power, increase the risk of false-positive findings, and limit the generalizability of results. Given the retrospective nature of the study and the strict inclusion/exclusion criteria, patient recruitment was constrained. However, the two groups exhibit comparable baseline characteristics in key aspects, which partially mitigates the impact of small sample size. Additionally, the retrospective design introduces potential selection bias. The well-matched baseline characteristics between groups may reflect survivor bias, as patients who completed 1-year follow-up may represent a healthier subset. Furthermore, treatment allocation was not randomized, and unmeasured confounders such as socioeconomic status, healthcare access, and patient motivation may have influenced outcomes. Second, the one-year follow-up period is insufficient to evaluate long-term treatment effects. Third, we did not measure peripheral B-cell counts or serum cytokine levels, which would have provided valuable mechanistic insights into the relationship between B-cell depletion and imaging changes. Fourth, key clinical metrics, including dynamic lesion volume changes (new/enlarging T2 lesions, gadolinium-enhancing lesions) and annualized relapse rates, were not analyzed. This unavailability is primarily due to the retrospective nature of the study: some patients' clinical records lacked detailed documentation of relapse events during the follow-up period, and the imaging protocol for a subset of participants did not include gadolinium-enhanced sequences, which precluded consistent assessment of contrast-enhancing lesions across the entire cohort. The absence of these conventional MRI and clinical disease activity markers limits our ability to definitively distinguish whether the observed imaging changes reflect true neuroprotective effects or nonspecific effects of inflammatory suppression. Without data on new lesion formation or relapse rates, we cannot rule out that the improvements in brain structure and function are secondary to reduced inflammation, rather than direct neuroprotection. This highlights the need for future prospective studies that integrate these standard disease activity endpoints with multimodal MRI to clarify the nature of OFA's effects.

Future multicenter studies with larger patient cohorts and extended follow-up periods should incorporate serial MRI lesion monitoring and clinical relapse data to: (1) Validate thalamocortical FC as a predictor of treatment response. (2) Assess the utility of ILF FA as a biomarker for disability progression in patients undergoing OFA therapy. (3) Validate the value of combining multimodal MRI with peripheral biomarkers (e.g., cerebrospinal fluid or blood-derived) for bridging these distinct scales of pathophysiology.

## Conclusion

In conclusion, this exploratory longitudinal study provides preliminary neuroimaging evidence that OFA treatment may help preserve thalamocortical connectivity, white matter microstructure, and gray matter volume in patients with RMS over a one-year follow-up period. These findings support mechanistic hypotheses regarding the neuroprotective effects of OFA, which require further validation in larger-scale prospective cohorts.

## Data Availability

The original contributions presented in the study are included in the article/Supplementary material, further inquiries can be directed to the corresponding author.
